# Clinical Outcomes of Patients with Multiple Myeloma after Daratumumab Failure

**DOI:** 10.3390/life13091841

**Published:** 2023-08-31

**Authors:** Irene Zamanillo, Lucia Medina de Alba, Rodrigo Gil, Rosalia de la Puerta, Rafael Alonso, Ana Jimenez-Ubieto, Maria Teresa Cedena, Maria Calbacho, Rosa Ayala, Joaquin Martinez-Lopez

**Affiliations:** 1 Hematology Department, University Hospital 12 de Octubre, 28041 Madrid, Spain; 2 Hematology Department, University and Polytechnic Hospital, 46026 Valencia, Spain

**Keywords:** daratumumab, refractory, relapsed, multiple myeloma, anti-CD38 monoclonal antibodies

## Abstract

Anti-CD38 monoclonal antibody (MoAB) therapy has significantly improved the prognosis of patients with multiple myeloma. However, not all patients sustain durable responses. We aimed to describe the natural history of patients relapsed or refractory (R/R) to CD38 MoAB therapy. We performed a single-center, retrospective analysis of the clinical characteristics and outcomes of 81 patients with multiple myeloma who progressed after treatment with daratumumab. Our cohort was heavily pretreated, with a median of two lines prior to daratumumab and only 17 patients received daratumumab as a first line. A total of 38.2% had received a previous autologous stem cell transplantation (ASCT), and 61.7% had received both an immunomodulatory drug (IMID) and a proteasome inhibitor (PI). The median overall survival (OS) was 21 months for the global cohort but it decreased to 14 months for triple-class refractory patients and 5 months for penta-refractory patients. Most of the patients (83.9%) received treatment after daratumumab progression, in many cases with second generation IMID or PI, but seven patients were treated with anti-BCMA therapy and three patients received CART therapy within a clinical trial. In conclusion, patients R/R to daratumumab represent an unmet clinical need with poor prognosis and in need of incorporation of new treatments.

## 1. Introduction

Daratumumab and isatuximab are clinically effective monoclonal antibodies (MoABs) that target CD38, a transmembrane glycoprotein receptor expressed at high levels on plasma cells [1,2]. They have demonstrated efficacy both as single agents [3,4] and in combination with other anti-myeloma drugs [5,6,7], which has led to their approval by the Food and Drug Administration (FDA) and the European Medicines Agency (EMA) for the treatment of multiple myeloma (MM), as a first-line therapy and for relapsed or refractory (R/R) MM. However, not all patients achieve long-term clinical benefits from daratumumab and isatuximab, and the majority will ultimately progress while on treatment or relapse after the therapy. While the mechanisms of resistance remain poorly characterized, they have been related to the downregulation of CD38 expression and to immune-mediated effects [1]. Studies describing the natural history of patients with daratumumab-R/R disease are scarce, but they show that patients have poor prognosis and shorter responses to subsequent treatment. Different papers have reported an overall survival (OS) of 6.6–11.2 months and progression-free survival (PFS) during the subsequent line of treatment of only 2.3–3.4 months in patients with advanced R/R multiple myeloma [8,9].

Patients progressing after CD38 MoAB-based therapies are also frequently resistant to other commonly used anti-myeloma agents such as lenalidomide and bortezomib. Patients who are “triple refractory” (refractory to a Proteasome inhibitor (PI), an Immunomodulator (IMID) and an anti-CD38 MoAB) are a particularly high-risk population, and have worse outcomes compared to patients exclusively resistant to CD38 MoABs, with shorter OS ranging from 3.5 to 9.2 months [8,9]. This can be attributed, at least in part, to both more aggressive diseases, and to the difficulties in identifying an effective salvage treatment. In addition, patients who are triple-refractory are typically “unfit”, as they have experienced several relapses with consequent functional decline.

Patients with triple-class refractory MM represent an unmet clinical need, and more studies are warranted to determine the optimal therapeutic approaches in this setting. Fortunately, new developments such as antibody–drug conjugates, T-cell-directed therapies, and novel small molecules may have an important impact in the future treatment of this multidrug-resistant population [10].

To better understand the clinical features and the outcomes of this group of patients, we conducted a single-center, retrospective study of patients with refractory or relapsed MM after treatment failure with daratumumab.

## 2. Materials and Methods

We included 81 relapsed/refractory MM patients after daratumumab treatment at the Hospital Universitario 12 de Octubre in Madrid, Spain. Eligible patients received a variable number of cycles of daratumumab in monotherapy or in combination with other agents. Patients who discontinued treatment before re-evaluation were also included in this analysis as real-world data. The study was approved by the Hospital 12 de Octubre Institutional Review Board.

We collected clinical data regarding baseline patient (age, and sex) and disease characteristics (year of diagnosis, staging, high-risk cytogenetics, renal function, bone lesions and extramedullary disease at diagnosis). High-risk cytogenetics were defined as t(4;14), t(14;16), or del 17p. Patients refractory to to a PI, an IMID and an anti-CD38 MoAB were considered triple-refractory. Patients refractory to two PIs, two IMIDs and an anti-CD38 MoAB were considered penta-refractory. We gathered clinical information regarding treatment response, previous and subsequent treatments, patient status and survival. Response assessment and survival data were evaluated according to the International Myeloma Working Group [11]. A partial response or better was considered an objective response. In the subgroup of patients with R/R MM after anti-CD38 MoAB treatment who received further therapy, we determined which agents were more frequently used and their influence on OS.

**Figure 1:**
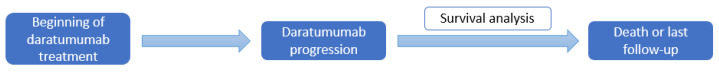
Survival was measured from the moment the patients became daratumumab R/R.

All data were compiled in an Excel spreadsheet and IBM SPSS (v25.0) was used for all the statistical analysis. We used the chi-square test to compare categorical variables. Logistic binary regression was used for multivariable analysis. Survival analysis was performed using the Kaplan–Meier method and comparisons between groups were assessed using the log-rank test. Two-sided *p* values of <0.05 were considered statistically significant. OS was measured from the moment of progression on daratumumab until the date of death or last follow-up for most analyses (Figure 1). OS for triple-class refractory or penta-refractory patients was calculated from the moment the patients met the criteria to be considered triple-class refractory or penta-refractory until death or last follow-up.

## 3. Results

### 3.1. Clinical Characteristics

The median age of patients at diagnosis of MM was 66 years (Interquartile Range (IQR) 55.5–73), and 55.6% were female. Extramedullary disease was present in 22.2% of patients at diagnosis and in 27.1% prior to daratumumab therapy. A total of 28.3% presented with high-risk cytogenetics (21% del17p, 3.7% t(4;14) and 4.9% t(14;16)) at diagnosis. Most of the patients had been pre-treated; only 17 patients (21%) received daratumumab as a first-line therapy. The median number of previous lines was 2 (range 0–8) and median time from diagnosis to daratumumab treatment was 30 months (IQR 9-63). A total of 38.2% of the patients had received prior autologous hematopoietic stem cell transplantation (ASCT), 66.7% had been previously treated with immunomodulators (IMID), 72.8% with proteasome inhibitors (PI) and 61.7% with both. Most patients received daratumumab in combination with other anti-myeloma agents, more frequently with carfilzomib (KDd 23.4%), with bortezomib (DVd 18.5%), and with melphalan and prednisone (D-VMP 16.0%). An amount of 22.2% received daratumumab in monotherapy or with corticosteroids. The main baseline characteristics of the patients are summarized in Table 1. First-line treatments for all the patients are outlined in Table 2.

### 3.2. Outcomes

Regarding the natural history of patients with refractory/relapsed MM after CD38 MoAB therapy, the median OS after daratumumab progression was 21.0 months (95% C.I. 10.7–31.2) for the global cohort and median time to progression on treatment with anti-CD38 MoAB was 7 months (IQR 3-20). The best response achieved during treatment with anti-CD38 MoAB was complete response (CR) with negative measurable residual disease (MRD) for 13 patients (22.3%), CR with positive MRD for 15 (18.5%) and partial response (PR) for 34 patients (41.9%). A total of 19 patients (23.5%) were refractory to daratumumab.

Progression to daratumumab as a first-line treatment was observed in 17 patients (21% of this series). Most of them (14, 82.3%) were considered non-eligible for ASCT and received D-VMP or DRd. The median age of this group at diagnosis was 73 years (IQR 69-76), and 58.8% were female. The proportion of cases with high-risk cytogenetics (11.7%) and extramedullary disease (25%) was not statistically different from patients treated with daratumumab in later lines. Median time to progression on daratumumab treatment was 15 months (SD 11.6), the best response achieved was CR with negative MRD in three (17.6%) patients, CR with positive MRD in five (29.4%), partial response in eight (47.1%) and one patient was refractory (5.3%). Although responses were better when patients received daratumumab as a first-line agent, there were no significant differences compared to patients who were treated with daratumumab later on (Table S1 Supplementary Material).

**Figure 2:**
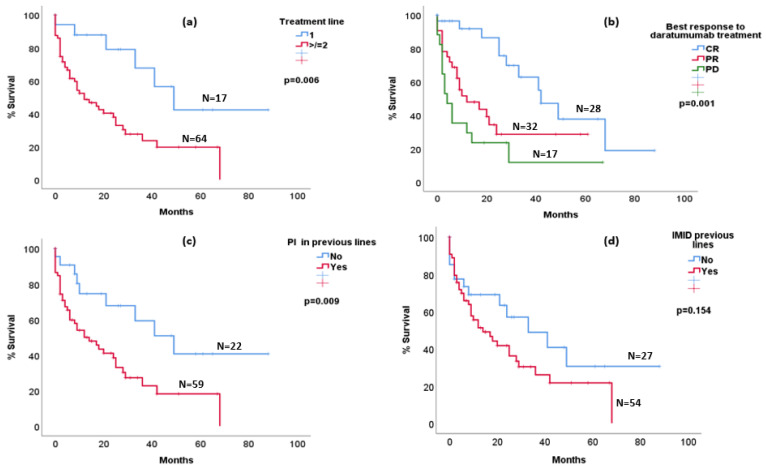
Kaplan–Meier curves representing survival after daratumumab progression in (**a**) patients who received daratumumab in first line vs. second or later lines; (**b**) patients who achieved complete response vs. patients who achieved partial response vs. patients who did not achieve any response to daratumumab therapy (CR—Complete Response; PR—Partial Response; PD—Progressive Disease); (**c**) patients who received treatment with PI (PI—Proteasome Inhibitors) vs. patients who did not receive treatment with PI before daratumumab progression; (**d**) patients who received treatment with IMID (IMID-Immunomodulator) vs. patients who did not receive treatment with IMID before daratumumab progression.

Survival after daratumumab progression was better in patients treated with an anti-CD38 MoAB as a first line compared to those who received daratumumab in later lines of treatment (median OS 49 vs. 12 months; *p* = 0.006) (Figure 2a). Patients treated with a PI before daratumumab progression showed worse outcomes compared to those who had not been exposed to a PI before (median OS 12 months vs. 49 months; *p* = 0.009) (Figure 2c). This difference was not statistically significant for patients with previous IMID (median OS 14 months vs. 33 months; *p* = 0.154) (Figure 2d).

The depth of response to the treatment line containing a CD38-targeted MoAB was predictive of survival (*p* = 0.001). The median OS was 42 months (95% C.I. 25.8–58.1) for patients with CR (*n* = 28), 12 months (95% C.I. 0–24.2) for patients with PR (*n* = 32) and 4 months (95% C.I. 0.7–7.2) for patients with no response (*n* = 17) (Figure 2b).

Sixty-eight patients (83.9%) received further treatment for relapsed or refractory MM after anti-CD38 MoAB therapy (median 2, range 1–5). The salvage regimens employed immediately after daratumumab progression were greatly variable. Most patients received pomalidomide-based regimens (32 patients, 47%), lenalidomide-based regimens (11 patients, 16.1%) or carfilzomib-based regimens (6 patients, 8.8%). Interestingly, seven patients received anti-BCMA therapy and three patients were treated with BCMA-targeting CAR-T therapy within a clinical trial. A total of 61.7% of the patients who received further treatment achieved a partial response or better to the next line of therapy and median PFS to this line of treatment was 8 months. The median OS for patients who received treatment after daratumumab progression was 25 months (95% C.I. 16.1–33.8). Among the 13 patients who did not receive further therapy after daratumumab progression, survival was very poor (median OS 0 months; range 0–8), and most of the patients (71.4%) died due to disease progression. We found no differences in PFS or OS depending on the type of salvage treatment. The treatment regimens and response rates to the treatment line after daratumumab progression are described in Table 3.

Within the cohort, 41 (50.6%) patients met criteria for triple-class refractoriness, with a median time from diagnosis of 52 months (SD 40.2), and with a median number of previous lines of 2 (range 1–8). These patients presented an OS of 14.0 months (95% C.I. 5.8–22.1). Regarding treatment response to subsequent lines, 8 patients (19.5%) reached complete response with subsequent lines of treatment, 13 patients (31.7%) reached partial response and 7 patients (17.0%) did not respond to any of the following lines of therapy. Finally, 13 patients did not receive further treatment after progressing to an IMID, a PI and an anti-CD38 monoclonal antibody. In our cohort, only 24 patients became penta-refractory. The median time from diagnosis to penta-refractoriness was 61 months (SD 37.2) and OS after penta-refractoriness was only 5 months (95% C.I. 0–13.7).

## 4. Discussion

Papers describing the natural history of R/R MM patients are scarce [12,13,14,15,16]. Here, we describe the clinical characteristics and survival of patients relapsed or refractory after daratumumab therapy. In our cohort, we observed an OS after daratumumab progression of 21.0 months. These results are better than the ones previously published, which could be explained by the incorporation of daratumumab to the first line of treatment and to the development of new therapies with novel mechanisms of action which can be used in salvage regimens.

Other papers have described the natural history of advanced MM patients after progression to anti-CD38 MoAB therapy, and they have reported a median OS of 6.6–11.2 months [8,9]. Importantly, these studies were conducted in multi-refractory patients and they did not include patients treated in first line. Therefore, the improved survival in our series could be explained by the integration of daratumumab in progressively earlier lines of treatment. In our cohort, patients treated in first line presented significantly better outcomes, with a median OS of 49 months compared to those treated as a second line or later, with an OS of 12 months. Notably, OS in multi-refractory MM patients in our series was very similar to the available data in the literature. The worsened survival in these patients can be attributed to both patient-dependent factors, including populations with several previous lines of treatment and limited choices for subsequent therapy, and to disease-dependent factors such as aggressive diseases, which develop resistance to new therapeutic agents with different mechanisms of action.

In this line, patients previously exposed to PI presented worse survival in our series (median OS 12 months), and patients previously exposed to lenalidomide showed a trend towards worsened outcomes (median OS 14 months), although the difference was not statistically significant probably due to low statistical power. Gandhi et al. [9] described survival in MM patients progressing to daratumumab according to the refractoriness level. They considered patients refractory to a PI, an IMID, and an anti-CD38 MoAB as triple-refractory, and patients refractory to two PIs, two IMIDs, and an anti-CD38 MoAB as penta-refractory. In their study, survival decreased progressively in the more refractory groups (11.2 months for not triple refractory, 9.2 months for triple refractory and 5.6 months for penta-refractory). There were similar findings in our cohort, with OS of 24 months for non-triple-refractory patients, 14 months for triple-refractory patients and 5 months for penta-refractory patients. The lowering survival for patients with a higher degree of refractoriness can be justified both by the limited options for effective therapy for these patients and could also reflect a more aggressive disease with a worse response to different agents.

In this aspect, another factor that could play a role in improving patient survival is the emergence of new agents with innovative targets. In our cohort, a significant percentage of patients (83.9%) received treatment after progressing on daratumumab treatment. We analyzed the line of treatment immediately after daratumumab progression, as we considered that this could have an important impact on patient outcome. The majority of patients received treatment with IMID or PI, often second-generation drugs, and it is worth noting that 7.3% of these patients were treated with anti-BCMA therapy and 4.4% were treated with CAR-T therapy, most within clinical trials. Patients who were treated after anti-CD38 MoAB therapy failure showed longer survival, (OS 25 vs. 0 months; *p* < 0.001). Gandhi et al. [9] also described lower survival for patients who did not receive treatment after anti-CD38 MoAb progression (OS 9.3 vs. 1.3 months).

Interestingly, we found a relationship between the depth of response obtained with daratumumab treatment and the overall survival after progression to this line in our series. This probably reflects the high-risk biology of these MM patients, which may not always be reflected by high-risk cytogenetics. In addition, it is possible that there are cross-resistance mechanisms which impact the effectiveness of several therapeutic families.

The present study has several limitations, including its retrospective nature and its moderate number of patients, which may lead to a low statistical power in detecting differences between groups Also, the population was heterogeneous regarding age, line of treatment, prior therapies received and response achieved. The strengths of the study include the real-life analysis of a cohort of patients with resistant or relapsed MM after treatment failure with anti-CD38 MoABs using a comprehensive dataset from a single center, which is representative of the highly variable MM population that is treated at a tertiary institution.

In conclusion, anti-CD38 MoAb-resistance is an unmet clinical need with poor prognosis and limited treatment options, even in patients treated in first line, although the incorporation of new targets and treatments has a positive impact. Studies describing mechanisms of resistance to anti-CD38 MoABs are essential to better understand the biological characteristics of the disease and to assist physicians in providing the best clinical care possible for these patients.

## Figures and Tables

**Table 1:** Main characteristics of the cohort. The table displays the count and percentage of patients with a specific characteristic among the total number of patients (first column), patients who received daratumumab as a first- or second-line treatment (second column) and patients who received daratumumab in later lines of treatment (third column).

Characteristics	All Patients *n* = 81 (%)	L 1–2 *n* = 39 (%)	L ≥ 3 *n* = 42 (%)
Age at diagnosis—median (IQR)	66 (55.5–73)	70 (59–75)	64.5 (50.7–72)
Females	45 (55.6)	20 (51.3)	25 (59.5)
Extramedullary disease at diagnosis	18 (22.2)	9 (23.1)	9 (21.4)
High-risk cytogenetics at diagnosis	23 (28.3)	10 (25.6)	13 (31)
IMID previous lines	54 (66.7)	15 (38.5)	39 (92.9)
PI previous lines	59 (72.8)	18 (46.2)	41 (97.6)
IMID and PI previous lines	50 (61.7)	12 (30.8)	38 (90.5)
Previous ASCT	31 (38.2)	9 (23.1)	21 (50)
Nº previous treatment lines: median (range)	2 (0–8)	1 (0–1)	3 (2–8)
Daratumumab containing regimen:			
Monotherapy with daratumumab	18 (22.2)	2 (5.1)	16 (38)
D-VMP	13 (16)	13 (33.3)	0
DKd	19 (23)	7 (17.9)	12 (28.5)
DVd	15 (18.5)	7 (17.9)	8 (19)
DPd	5 (6.1)	2 (5.1)	3 (7.1)
DRd	5 (6.1)	4 (10.2)	1 (2.3)
Other	6 (7.4)	4 (10.2)	2 (4.6)

ASCT: autologous stem cell transplant; L: line of treatment containing an anti-CD38, MoAb. *n*: number of patients; Daratumumab-containing regimen: D-VMP: Daratumumab, Bortezomib, Melphalan and Prednisone; DKd: Daratumumab, Calfilzomib and Dexamethasone; DVd: Dartumumab, Bortezomib and Dexamethasone; DPd: Dartumumab, Pomalidomide and Dexamethasone; DRd: Daratumumab, Lenalidomide and Dexamethasone.

**Table 2:** Regimens used in first-line therapy for the patients.

Frist Line Therapy (*n* = 81)	
	D-VMP: 12 (14.8%)
Regimens with daratumumab	DVd: 4 (5%)
	DRd: 1 (1.2%)
	RD: 8 (9.9%)
Regimens with IMID	VRD: 15 (18.5%)
	VTD: 13 (16%)
	KRD: 1 (1.2%)
	VMP: 11 (13.6%)
Regimens with bortezomib	VD: 4 (5%)
	V-bendamustine-prednisone: 3 (3.7%)
	Chemotherapy VBCMP/VBAD: 3 (3.7%)
Others	MP: 1 (1.2%)
	KMP: 2 (2.4%)

D: daratumumab; K: carfilzomib; M: melphalan; P: prednisone; R: lenalidomide; T: thalidomide; V: bortezomib; VBCMP/VBAD: vincristine, carmustine, melphalan, ciclophosphamide and vincristine, carmustine, doxorubicin and dexamethasone.

**Table 3:** Treatment regimens used after daratumumab progression and response rates. Representation of the number and proportion of patients who achieved a determined response for each therapeutic scheme.

Treatment Regimen (*n* Patients)	Complete Response	Partial Response	No Response
Pomalidomide-based treatment:			
PomCyDex (18)	6 (33.3%)	5 (27.7%)	7 (38.8%)
PomDex (11)	3 (27.2%)	2 (18.1%)	6 (54.5%)
PVD (3)	1 (33.3%)	1 (33.3%)	1 (33.3%)
Lenalidomide-based treatment:			
Rd (10)	5 (50%)	4 (40%)	1 (10%)
KRd (1)	0	1 (100%)	0
Carfilzomib-based treatment:			
KyCyDex (3)	0	1 (33.3%)	2 (66.6%)
Kd (3)	1 (33.3%)	1 (33.3%)	1 (33.3%)
Anti-BCMA:			
Belantamab (2)	0	1 (50%)	1 (50%)
Talquetamab (2)	0	1 (33.3%)	1 (33.3%)
Teclistamab (1)	0	0	1 (100%)
Not specified (2)	1 (50%)	1 (50%)	0
CAR-T (3)	2 (66.6%)	1 (33.3%)	0
Others (9)	1 (11.1%)	3 (33.3%)	5 (55.5%)

Kd: Carfilzomib-Dexamethasone; KRd: Carfilzomib–Lenalidomide–Dexamethasone; KyCyDex: Carfilzomib–Cyclophosphamide–Dexamethasone; PomCyDex: Pomalidomide–Ciclophosphamide–Dexamethasone; PomDex: Pomalidomide–Dexamethasone; PVD: Pomalidomide–Bortezomib–Dexamethasone; Rd: Lenalidomide–Dexamethasone.

## Data Availability

The data to support these findings are available upon request due to privacy restrictions.

## Supplementary Materials

The following supporting information can be downloaded at https://www.mdpi.com/article/10.3390/life13091841/s1: Table S1: Representation of the number and proportion of patients who achieved certain response to daratumumab treatment as a front line or in later lines.
