# Supplementary material for: Clinical Outcomes of Patients with Multiple Myeloma after Daratumumab Failure
Source: Life (Basel). 2023 Aug 31;13(9):1841. doi: 10.3390/life13091841 (PMC10532632; doi:10.3390/life13091841)
Supplement: Supplementary file 1 [file life-13-01841-s001.zip › life-2438009-supplementary.pdf]

	Complete response	Partial response	No response
Patients treated with daratumumab in first line (n=17)	8 (47%)	8 (47%)	1 (5.9%)
Patients treated with daratumujab in second line or later (n=64)	20 (31.2%)	26 (40.6%)	18 (28.1%)

Table 1 supplementary material. In each line is represented the number and proportion of patients who achieved certain response to daratumumab treatment as a front line (first row) or in later lines (second row).
